# Veterinary Dentist1Read before the Twelfth Annual Meeting of the New York State Veterinary Medical Society, Brooklyn, September 9 and 10, 1902.

**Published:** 1902-12

**Authors:** R. W. Ellis

**Affiliations:** New York City


### "VETERINARY DENTISTRY."[1]

By R. W. Ellis, D.V.S.,

NEW YORK CITY.

Solomon, I think it is, who is credited with having said "There is nothing new under the sun." Whether this be true or not, we are daily forced to look at old things from new points; and in this paper I am endeavoring to view "veterinary dentistry" from a different point of view from the one which, it seems to me, most of us view it. We have regarded it too much, I fear, as something that needs no special attention from us as veterinary practitioners beyond the point of "fixing a horse's mouth" when called upon to do so by coachman or trainer, and solely because they ask us to do so, not, as in other operations, because we decide it is the thing to be done after examination of the subject.

The cause, I think, for the suggestion to operate upon the teeth, usually emanating from the source mentioned instead of (as it should be) from the practitioner, is that we do not pay a sufficient amount of attention to the horse's mouth. The result of this inattention on our part has made this particular branch of veterinary surgery a prey to empiricism and a menace to our practice laws in States where they exist. Having thus tried to establish what seems to me a fair justification for introducing this topic, and engaging your interest, I will say, from Solomon's viewpoint, that I am not attempting to teach the members of this Association anything new, but simply wish to review with you, briefly, an old subject, which impresses me with greater importance as I look into it more closely, with the hope of provoking a discussion and learning something therefrom. Leaving aside the structure

[1] Read before the Twelfth Annual Meeting of the New York State Veterinary Medical Society, Brooklyn, September 9 and 10, 1902.

of the teeth and all tedious minutiæ (data upon which is as accessible to all present as it is to me, and its repetition here tiresome), let us at once proceed to briefly review the horse's mouth relative to his teeth in the normal state, before touching upon the abnormalities which, it is my purpose to show, would either not exist or be present in a much modified form if we did our duty at all times in regard to making examinations of the horse's mouth. I will endeavor to qualify this statement at another point in this paper. The number of teeth ranges from thirty-six to forty-four, forty being the number usually considered normal and regular in the horse, consisting of twenty-four molars, twelve incisors, and four tusks or canine teeth, commonly called bridle teeth. The absence of the four last named, being considered regular and normal in the mare, makes her total thirty-six. These teeth, however, are sometimes present in the mare as she advances in age. In either sex we may find in addition to those already mentioned four additional supplementary premolars or vestigials; their situation, when present, being directly in front of the first premolar in both the upper and lower jaws. I have seen but one mouth in which the vestigial premolar was present in the lower as well as the upper jaw. In this case it was present on one side only, in the lower jaw; and I have every reason to believe that their existence in the lower jaw is extremely rare. Discussion may throw some light on it. Just why the vestigial premolars, as we find them in the horse, are in irregular condition, existing in some subjects and not in others, I am not prepared to state positively. One theory is advanced that they do exist regularly prior to the replacing of the "milk-teeth" by the permanent teeth, they forming the number of the first set, and are in most instances crowded out by the permanent teeth, but occasionally being retained; hence, we find them as an irregular condition. I am inclined to accept this theory, especially as we notice that other animals, carnivora, swine, etc., have four premolars instead of three, making seven in all in each jaw, instead of six as in the horse. Indeed, naturalists insist upon four premolars in the horse, counting the vestigials as the first; and what we call the first they term the second premolar, because if the vestigial does not exist they contend that it has been crowded out with the shedding of the milk-teeth—a condition which might readily occur when we take into consideration that the premolars in the horse are vastly different from those in other species of animals in that in the case of the horse they are the largest teeth in the whole row, in contradistinction to other animals, including man, in which they are somewhat insignificant as compared with those succeeding them. Dame Nature seems to have concluded that the character of the horse's food demands that he have three good stout premolars rather than four indifferent ones, the fourth one remaining only as a trace (as its name implies) of a tooth which positively was developed in the prehistoric horse. Not infrequently in the horse we find the last molar to be the smallest in the row, especially in the lower jaw, a contrary condition to that of the carnivora and man. The molars of the lower jaw in the horse are, approximately, about two-thirds the width of those of the upper jaw. There is also considerable disparity in the width of the two jaws, the upper being much wider than the lower jaw; and when the mouth is closed, so that the upper and lower incisors sit squarely over each other, the lower molars do not cover more than about an eighth of an inch on each side of the inner border of the upper molars. The amount is not the same over the entire length, however, varying with the degree of curvature of the upper jaw; so that the horse can only grind on one side of the jaws at one time, during which the incisors are not at all in contact. In a normal mouth the grinding surfaces of the lower molars should bevel slightly from above to below and from within outward, and the opposite condition exist in the upper molars; and there should be nothing existing either on the grinding surfaces or on their borders to prevent them from passing over the full width of their wearing plate while in contact over their entire length. Any condition other than this is abnormal. Following are some examples: First, and the most common condition that we meet, are sharp spiculæ, which result from the extremities of the rib-like folds on the outer surfaces of the teeth being gradually drawn out to sharp points by the two bevelled plates passing over each other during the process of grinding food; the harder materials in the tooth-formation persisting, while the less harder wear away. These spiculæ, if not removed, lacerate the inner sides of the cheeks as a result of pressure from without by the cheek-piece of the bridle, and themselves become the cause of further trouble by preventing the two plates of the upper and lower jaws from passing completely over each other, thus restricting the grinding surface. This in turn causes these "spiculæ," small and comparatively innocent at first, by lack of contact with opposing surfaces, to increase in length and continue to more materially interfere with the proper mastication of food; and as they increase in length their base also becomes broader, and they continue to restrict more and more the lateral movements (diduction) which the jaws describe in crushing the food. This, if allowed to go on indefinitely, might be responsible for the very grave condition described by Girard and H. Bouley as "bevecing of the molars;" in which condition the tables of the molars, instead of meeting each other on an almost horizontal plane, are sometimes worn down obliquely, so that they become almost parallel to the median plane and overlap each other like the blades of a pair of shears. This obliquity of the wearing surfaces enables the external side of the superior molars and the internal side of the inferior to frequently acquire an enormous length, even to the extent of injuring the parts around the crowns. The French authors give this condition the name of "molaires en ciseaux," from their resemblance to a pair of shears. I do not wish to be understood as claiming that this grave condition just described is always or only due to neglected spiculæ, nor that neglected spiculæ would always lead to such an exaggerated abnormality; but that it is possible for it to result from neglected spiculæ, if they occur early in the animal's life, by restricting the movements of the jaw across each other, and gradually encouraging the upper jaws to spread outward and contracting the lower jaws. This, if carried on for a lifetime, would so change the direction of the teeth that instead of their grinding surfaces meeting the sides would meet and sharpen out in the manner described. But the direction of the teeth resulting in this abnormal relation and consequent condition might, on the other hand, not be due to neglected spiculæ, but to having started their growth at an improper angle. Another somewhat common condition that we are brought in contact with is a projecting molar, resulting from the absence of the opposing molar and consequent lack of wear. I will not touch upon the diseases of the teeth in this paper, but have simply mentioned one or two abnormal conditions as compared with the normal state, and will now endeavor to back the statement that these abnormalities would not exist or be present in a much modified form if we did our duty in regard to examining the horse's mouth at all times, and gave it attention when we considered it required it, and not merely when a layman thinks that something should be done. I do not mean that we shall be so undignified and unprofessional as to ask men to allow us to attend their horse's teeth; far from it. That is done by another class of "gentry" whom you all know; but we can be careful to examine a horse's mouth as to the condition of his teeth when we are called upon to prescribe for him. We may be told that he is "off his feed," or "out of condition," or "isn't just himself," "does not drive up as he used to," etc., and it is our privilege then to examine him thoroughly, and there is no restriction placed upon his mouth. If we find that his teeth are in perfect condition it helps to our diagnosis by removing them as one of the possible causes, and if, on the other hand, they require attention, we learn that. You have probably all of you, at some time or other, found a so-called "subject to colic" to be suffering from irregular molars, and on correcting that condition had the satisfaction of seeing the horse enjoy the same freedom from that trouble that another horse would. I have. When examining horses for soundness we have the privilege of passing the hand over the surface of the molars just as we would pass it over any other part of the body. We need not condemn him because his teeth are slightly irregular, and we need not mention it in our report to our client, merely making a mental note of it for future use; but if there exist a molar decayed or one missing (not likely conditions, it is true, but possible ones, nevertheless), it is our duty to our client to know of their existence and to acquaint him of it. If we have found merely irregularities, we need not call his attention to it necessarily until after he has purchased, as it is a remediable condition; but we should then, by all means, acquaint him of the fact, and impress upon him the importance of this grist-mill (the mouth) turning out a product that is in proper condition to pass down into the laboratory (the stomach), where extracts are to be made from it for the requirements of the body. One or a dozen men cannot materially effect the condition of the horse's teeth of the country any more than one man can repress glanders, but if there was unity of action in that direction, very few mouths would escape observation for any length of time, and would not have opportunity of reaching the extreme conditions cited; and the horse owner, impressed with the importance of the subject and the interest taken in it by the professional man, would not be a prey to the empirical self-styled "veterinary dentist," who hopes by thus dubbing himself a specialist to impress the uninitiated and escape the law forbidding him to practice veterinary surgery without a license. I always insist upon my students at the college examining the molars as well as the incisors in examinations for soundness, with the hope that they will continue to do so in practice, and more and more appreciate and teach their clients to appreciate its importance, so that "veterinary dentistry," like other branches of medicine and surgery, may become prophylactic. Gentlemen, while I know that I have scarcely outlined the subject under the title of this paper, I will end it at this point, and trust that my shortcomings may be overlooked because I am considerate enough to *stop* somewhere. In fact, the paper would have been more properly entitled "The Horse's Mouth," as I have not dealt with a single procedure to overcome the abnormalities mentioned, which would be, more correctly speaking, "veterinary dentistry." I have purposely curtailed my paper, so as to leave the operative field untouched, as we have another paper on the same subject, which I have assumed will enter into it.

